# Use of the float-moor-crush approach for subtotal mid-segment collapse of a protruding aorto-ostial vein graft stent: a case report

**DOI:** 10.4076/1752-1947-3-8497

**Published:** 2009-09-08

**Authors:** Lieuwe H Piers, Gillian AJ Jessurun, Rutger L Anthonio

**Affiliations:** 1 Department of Cardiology, Thoraxcenter, University Medical Center Groningen, Hanzeplein, 9700 RB, Groningen, The Netherlands

## Abstract

**Introduction:**

Aorto-ostial stenting remains one of the most demanding and risky types of angioplasty to perform. We report a case outlining a creative solution for the reengagement of a protruding aorto-ostial stent.

**Case presentation:**

A 69-year-old Caucasian man was admitted to our hospital's coronary care unit with progressive unstable angina five years following coronary artery bypass grafting and three years after percutaneous coronary intervention of the graft. Several attempts to engage the protruding part of the aorto-ostial stent in the graft failed. A catheter was eventually floated towards the protruding part using a wire to moor the catheter to the stent through the side-strut. The proximal part of the protruding stent was subsequently crushed with a new stent. Stent patency was observed 12 months after the procedure was performed.

**Conclusion:**

Although careful cannulation of a aorto-ostial stent during repeat coronary angiography coupled with the placement of a guidewire and stent through the true stent lumen during repeat intervention remains the ideal approach for aorto-ostial in-stent restenosis, this case report confirms the feasibility of the side-strut stenting technique in reaching a long-term positive outcome.

## Introduction

Aorto-ostial stenting remains one of the most demanding types of angioplasty to perform. Anticipation of risks such as potential stent loss, imprecise or malposed stent delivery and stent recoil or collapse should guide the technical approach of the procedure [1]. Aorto-ostial stenting after bypass surgery adds an additional risk to the overall technical outcome as the anastomotic area may be vulnerable especially during the early post-surgical period. Restenosis or occlusion of the aorto-ostial stent may render appropriate access to the stent difficult.

Aorto-ostial stenting carries a significant risk and aortic manipulation should be minimized. We present a case that demonstrates a creative solution for reengaging a protruding aorto-ostial stent.

## Case presentation

**Figure 1:**
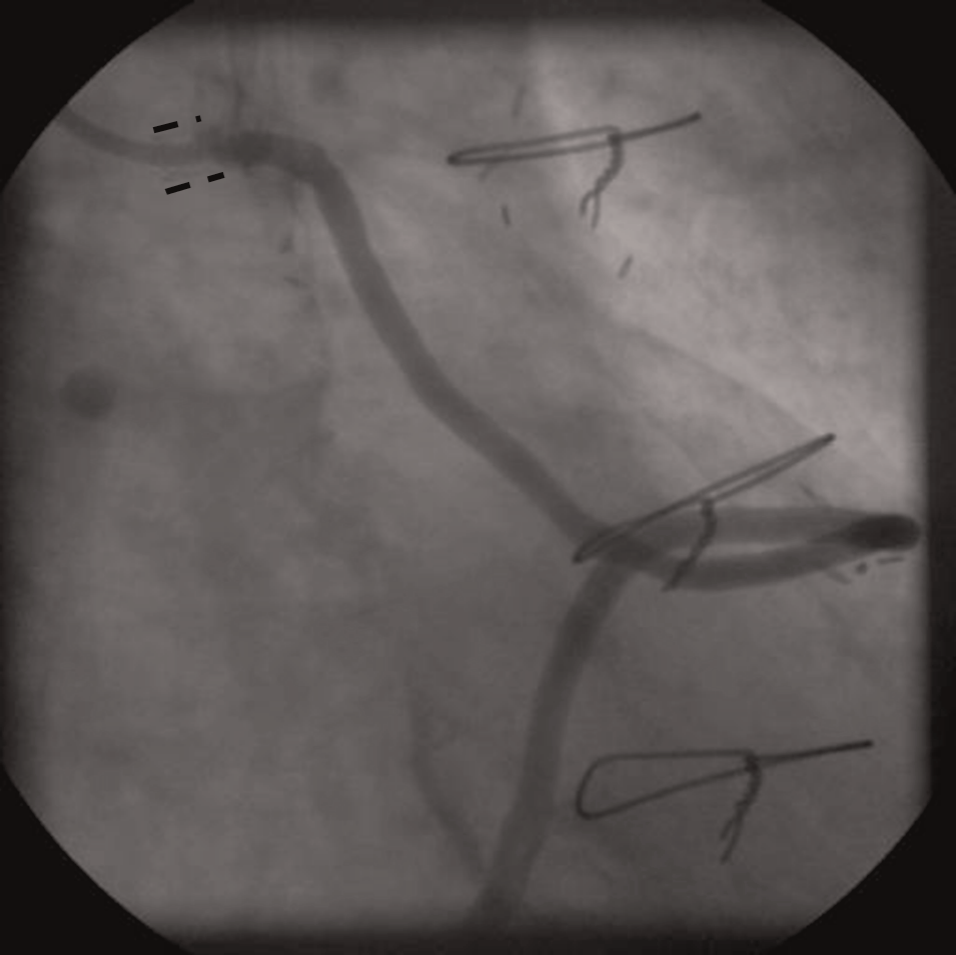
**Final result after aorto-ostial stenting of a vein graft showing significant protrusion of the stent (marked as = = =) into the aortic lumen**.

A 69-year-old Caucasian man was admitted to the coronary care unit with unstable angina five years following coronary artery bypass grafting (CABG) of his left internal mammary artery to his left anterior descending artery and a saphenous vein graft from the aorta to the diagonal, obtuse marginal branch and right descending posterior artery. Three years prior to presentation, the patient also underwent a percutaneous coronary intervention (PCI) of the stenotic ostium of the saphenous vein graft supplying the obtuse marginal coronary artery, with a Lekton motion 4.0 × 15 mm stent (Biotronik, AG, Bülach, Switzerland) at 16 atmosphere (Figure 1). One year after PCI, the patient still suffered from stable angina (NYHA III), and a second attempt to engage the stent in the vein graft ostium was performed. Unfortunately, the engagement was unsuccessful and the procedure was aborted. Another conservative approach was proposed and a PCI of the native left main and left circumflex artery was recommended in the event of progressive anginal complaints. Anti-anginal drug treatment was optimized with long acting nitrates.

**Figure 2:**
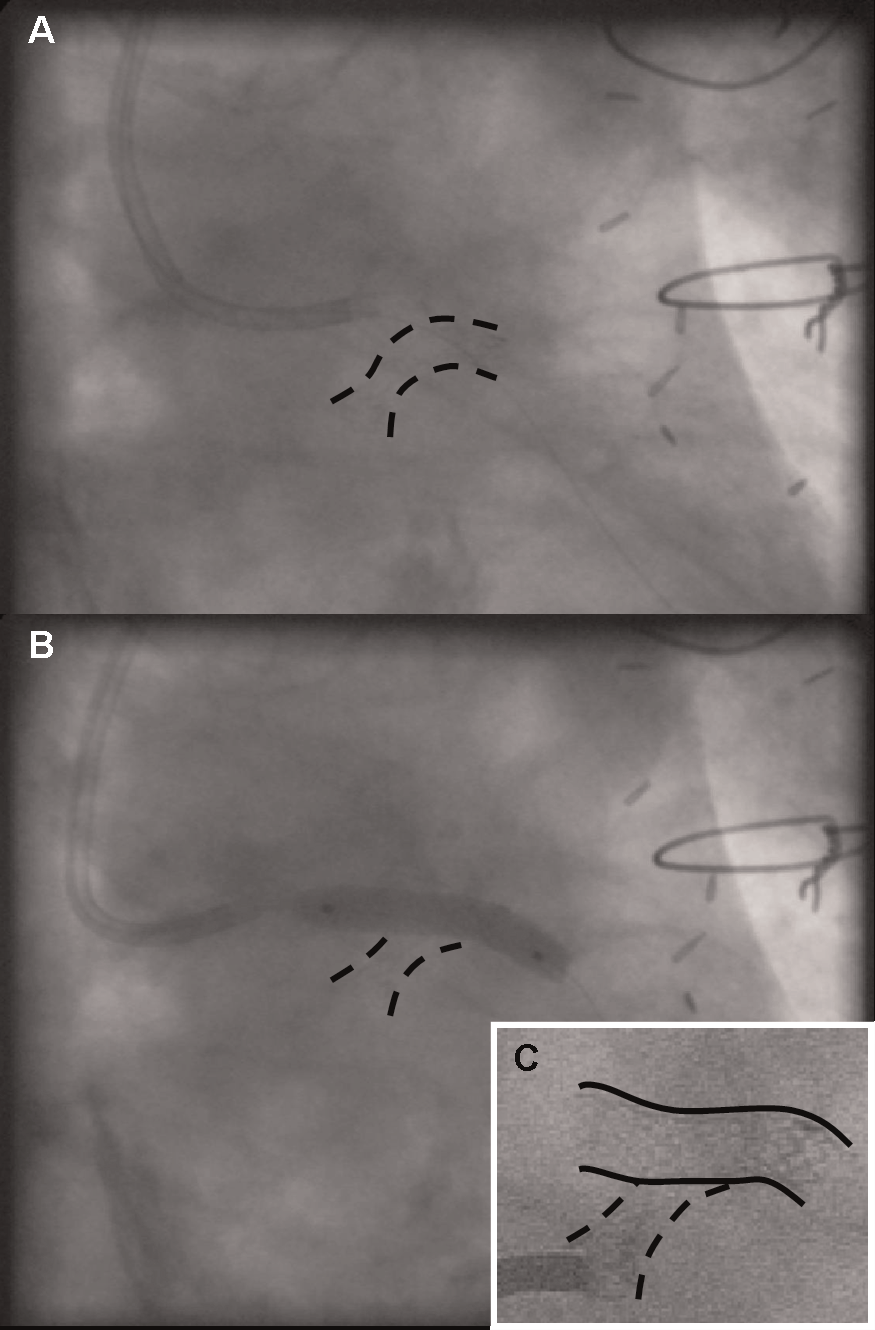
**Visualization of an aorto-ostial stenting procedure using the float-moor-crush technique**. The treating doctor chose to float a 3.5-mm coronary catheter towards the ostium stent. Subselective engagement of the vein graft shows the collapsed ostium stent (marked as = = =) at the mid-segment, visible as an hourglass aspect, and the mooring stage of the procedure **(A)**. After predilatation of the strut, a stent (====) was delivered and postdilated crushing the protruding stent at the aorto-ostial side; the crushing phase of the technique **(B)**. The crushed twin stent is visible as it points at an 80-degree angle of the new channel **(C)**.

**Figure 3:**
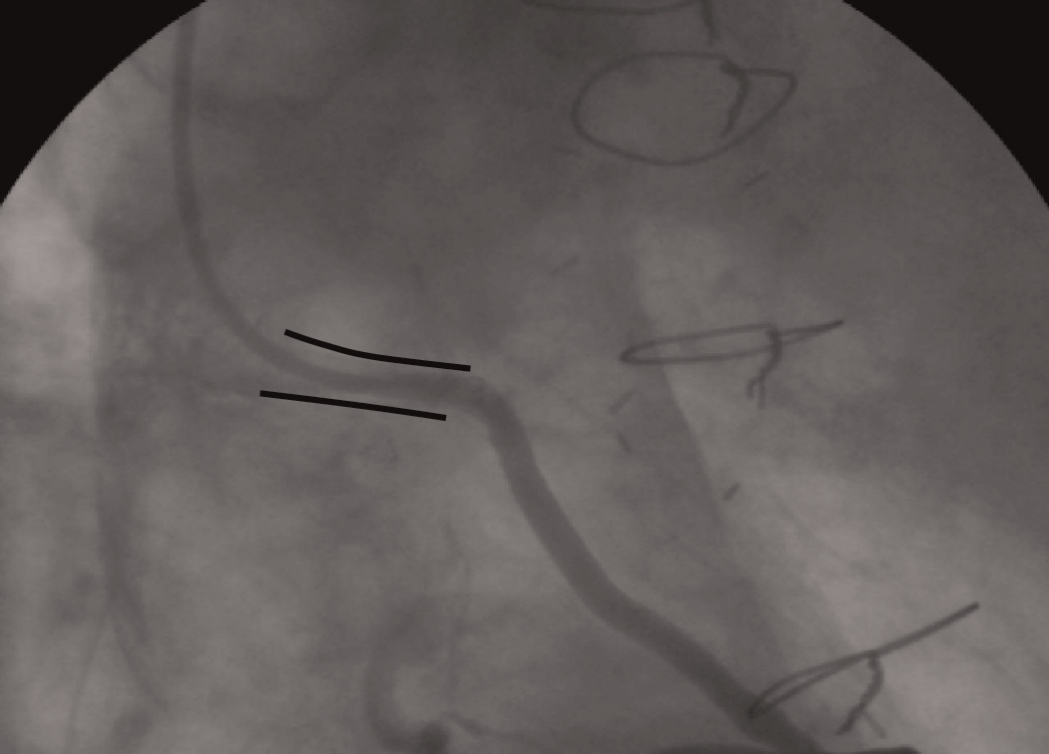
**Control angiogram of a vein graft 12 months after an aorto-ostial stenting procedure using the float-moor-crush technique shows deep intubation into a patent stent (marked as ====) and a good backflow**.

Three months later the patient was readmitted with unstable angina pectoris (NYHA IV). A third attempt to reengage the protruding stent in the vein graft ostium was discussed and planned. The protruding part of the aorta was long and pointed towards the aortic valve at an angle of about 45 degrees from the aortic wall. This made reengagement unsuccessful despite multiple attempts with the use of many 6F guiding catheters. Thereafter, an attempt to dilate the angulated and calcified left main coronary artery was aborted when rupture of the balloon complicated the procedure. Instead of trying to engage the stent by its true lumen, a maneuver that had repeatedly failed, the treating doctor chose to float a 3.5-mm coronary catheter (Medtronic) towards the protruding part of the stent until it stabilized. Subsequently, a rather supportive Pilot 150 wire (Guidant, Santa Clara, CA, USA) was used to moor the catheter to the stent through a side strut (Figure 2) and further advanced the wire to the peripheral portion of the graft. After a new channel was created by predilatation of the strut, a 3.5 × 15 mm Endeavour stent (Medtronic, Minneapolis, MN, USA) was delivered and post-dilated with a 4.0 × 15 mm balloon through the newly placed stent at 18 atmospheres, allowing the proximal part of the protruding stent to become crushed at the ostial side (Figure 3). Care was taken not to leave a large part of the stent protruding into the aorta (Figure 3). Clinical follow-up at 12 months showed stent patency after selective vein graft cannulation (Figure 3).

## Discussion

PCI of aorto-ostial coronary lesions is confronted by unique technical challenges not offered by other lesion subtypes [1]. These include poor guiding catheter support, difficult stent placement and incomplete stent expansion. These challenges are further enhanced during repeat interventions by poor visualization of the intra-aortic component of stent struts, non-coaxial guiding catheter engagement [2] and difficulty in placing the guidewire in the true stent lumen [3]. Furthermore, causal attempts at engaging the protruded stent and coronary ostium may deform the stent struts, making further coaxial guiding catheter engagement impossible.

Stenting for de novo aorto-ostial coronary lesions is recommended to prevent the strong elastic recoil inherent to these lesions and to reduce the risk of restenosis. Precise stent placement, however, is hampered by a lack of guiding catheter support and poor visualization during nonselective angiography. Despite these limitations, it is imperative that a stent should be placed across the coronary ostium with only 1mm to 2mm of the proximal stent segment protruding into the aorta to allow complete lesion coverage and minimize the risk of stent deformation during subsequent procedures. Similarly, when encountering a previously placed aorto-ostial stent, cautious catheter manipulation is essential to establish coaxial guiding catheter alignment without deforming the protruded stent struts. Placement of a guidewire in the true lumen of the stent is often not difficult after a coaxial guiding catheter has been engaged, but intricate guidewire techniques may be required if the protruded stent segment in the aorta is longer than a few millimeters.

As evident from this case, failure to achieve coaxial guiding catheter alignment despite cautious attempts with multiple guiding catheters, as well as the inability to advance the guidewire through the true stent lumen, may be the only signs of occult stent strut deformity. We managed these difficulties by placing the guiding catheter on top of the protruded stent and advancing a Pilot 150 (Guidant) guidewire though the struts of the intra-aortic stent segment. Serial balloon dilatations with sequentially larger, low-profile balloons were performed to widen the stent cell opening and dilate the collapsed stent. This facilitated the passage and placement of a second Endeavour stent across the lesion through the widened stent opening. PCI through stent struts has been reported for treatment of aorta-ostial in-stent restenosis [4,5]. Redo surgery, however, should be considered. The creative percutaneous approach employed in our patient was judged as safe and feasible. The decision for redo surgery would have probably been facilitated in the presence of more target lesions. Moreover, the presence of local adhesions and scar tissue growth during redo surgery remains a substantial limiting factor for clinical success. Current literature supports percutaneous intervention as a good clinical alternative to the surgical indications used in the past [6].

This case shows a side-strut stenting technique for complete aorto-ostial collapsed stent at the mid-segment. Side-strut stenting represents a modification of the culotte technique [7], and displaces the intra-aortic segment of the previously placed lateral stent, thus creating a new entry site into the coronary artery. Although careful cannulation of the aorto-ostial stent during repeat coronary angiography and placement of the guidewire and stent through the true stent lumen during repeat intervention remains the ideal approach for aorto-ostial in-stent restenosis, this report confirms the feasibility of the side-strut stenting technique in reaching a long-term positive outcome.

## Conclusions

The float-moor-crush approach may be described as a strategy combining both the side strut and culotte techniques, and should always be considered as a bail-out intervention in challenging aorto-ostial lesions.

## Abbreviations

CABG: coronary artery bypass grafting; NYHA: New York Heart Association; PCI: percutaneous coronary intervention.

## Competing interests

The authors declare that they have no competing interests.

## Consent

Written informed consent was obtained from the patient for publication of this case report and any accompanying images. A copy of the written consent is available for review by the Editor-in-Chief of this journal.

## Authors' contributions

GJ and RA analyzed the procedure describe in this manuscript. LP and RA wrote the manuscript. All authors read and approved the final manuscript.
